# (*E*)-2-[(3-Fluoro­phen­yl)imino­meth­yl]-4-(trifluoro­meth­oxy)phenol

**DOI:** 10.1107/S1600536810002989

**Published:** 2010-02-03

**Authors:** Yelda Bingöl Alpaslan, Gökhan Alpaslan, Ayşen Ağar, Şamil Işık

**Affiliations:** a Department of Physics, Faculty of Arts & Science, Ondokuz Mayıs University, TR-55139 Kurupelit-Samsun, Turkey; b Department of Chemistry, Faculty of Arts & Science, Ondokuz Mayıs University, 55139 Samsun, Turkey

## Abstract

The title compound, C_14_H_9_F_4_NO_2_, is a Schiff base which adopts the phenol–imine tautomeric form in the solid state. The H atom is located on the hydr­oxy O atom rather than on the N atom. This H atom is involved in a strong intra­molecular O—H⋯N hydrogen bond. The mol­ecule is almost planar, the angle between the benzene rings being 2.14 (13)°.

## Related literature

Schiff base compounds can be classified by their photochromic and thermochromic characteristics, see: Calligaris *et al.* (1972 ▶); Cohen *et al.* (1964 ▶); Hadjoudis *et al.* (1987 ▶). For Schiff base tautomerism,see: Şahin *et al.* (2005 ▶).
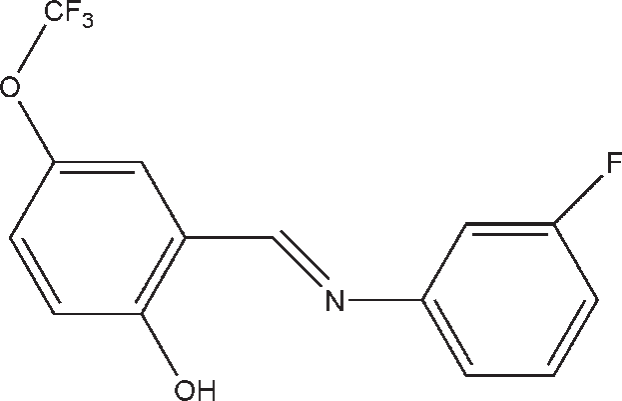


## Experimental

### 

#### Crystal data

C_14_H_9_F_4_NO_2_
*M* *_r_* = 299.22
Monoclinic, 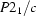

*a* = 14.223 (2) Å
*b* = 7.0894 (6) Å
*c* = 13.2479 (19) Å
β = 100.910 (11)°
*V* = 1311.7 (3) Å^3^
*Z* = 4
Mo *K*α radiation
μ = 0.14 mm^−1^
*T* = 296 K
0.72 × 0.34 × 0.07 mm

#### Data collection

Stoe IPDS-II diffractometer
Absorption correction: integration (*X-RED32*; Stoe & Cie, 2002 ▶) *T*_min_ = 0.929, *T*_max_ = 0.985
8512 measured reflections
2581 independent reflections
1261 reflections with *I* > 2σ(*I*)
*R*_int_ = 0.063

#### Refinement

*R*[*F* ^2^ > 2σ(*F* ^2^)] = 0.057
*wR*(*F* ^2^) = 0.133
*S* = 1.01
2581 reflections
222 parameters
66 restraints
H atoms treated by a mixture of independent and constrained refinement
Δρ_max_ = 0.12 e Å^−3^
Δρ_min_ = −0.14 e Å^−3^

### 

Data collection: *X-AREA* (Stoe & Cie, 2002 ▶); cell refinement: *X-AREA*; data reduction: *X-RED32* (Stoe & Cie, 2002 ▶); program(s) used to solve structure: *SHELXS97* (Sheldrick, 2008 ▶); program(s) used to refine structure: *SHELXL97* (Sheldrick, 2008 ▶); molecular graphics: *ORTEP-3 for Windows* (Farrugia, 1997 ▶); software used to prepare material for publication: *WinGX* (Farrugia, 1999 ▶).

## Supplementary Material

Crystal structure: contains datablocks I, global. DOI: 10.1107/S1600536810002989/si2237sup1.cif

Structure factors: contains datablocks I. DOI: 10.1107/S1600536810002989/si2237Isup2.hkl

Additional supplementary materials:  crystallographic information; 3D view; checkCIF report

## Figures and Tables

**Table 1:** Hydrogen-bond geometry (Å, °)

*D*—H⋯*A*	*D*—H	H⋯*A*	*D*⋯*A*	*D*—H⋯*A*
O1—H1⋯N1	0.91 (5)	1.81 (5)	2.618 (3)	146 (4)

**Fig. 1.**
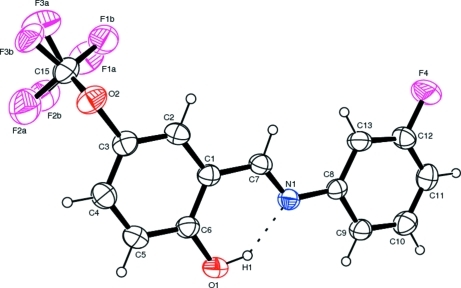
The molecular structure of (I), showing the atom-numbering scheme. Displacement ellipsoids are drawn at the 30% probability level and H atoms are shown as small spheres of arbitrary radii.

## supplementary crystallographic information

### Comment

Schiff bases have been extensively used as ligands in the field of coordination chemistry (Calligaris *et al.*, 1972). Schiff base compounds can be classified by their photochromic and thermochromic characteristics (Cohen *et al.*, 1964). These properties result from proton transfer from the hydroxyl O atom to the imine N atom (Hadjoudis *et al.*, 1987). There are two types of intramolecular hydrogen bonds in Schiff bases, which may be stabilized either in keto-amine (N-H···O hydrogen bond)(Şahin *et al.*, 2005) or phenol-imine (N···H-O hydrogen bond) tautomeric forms (Hadjoudis *et al.*, 1987). The present X-ray investigation shows that the title compound is a Schiff base and exists in the phenol-imine form in the solid-state.

An ORTEP-3 (Farrugia, 1997) plot of the molecule of (I) is shown in Fig.1. The N1-C7 bond length of 1.269 (5) Å is typical of a double bond. The dihedral angle between the C1-C7 and C8-C13 benzene rings is 2.14 (13)° and the compound is thus almost planar.  The compound shows a strong intramolecular hydrogen bond (O1-H1···N1).

### Experimental

The compound  (E)-2-((3-fluorophenylimino)methyl)-4-(trifluoromethoxy) phenol was prepared by reflux a mixture of a solution containing 2-Hydroxy-5-(trifluoromethoxy)benzaldehyde (0.010 g; 0.1 mmol) in 20 ml ethanol and a solution containing 3-Fluoroaniline  (0.0111 g; 0.1 mmol) in 20 ml ethanol. The reaction mixture was stirred for 1 hour reflux. The crystals of (E)-2-((3-fluorophenylimino)methyl)-4-(trifluoromethoxy) phenol for X-ray analysis were obtained from ethylacetate by slow evaporation (yield, 72%; m.p. 360-363 K).

### Refinement

The H atom bonded to O1 was refined freely. All other H atoms were placed in calculated positions and constrained to ride on their parent atoms, with C—H = 0.93 Å and *U*_iso_(H) = 1.2 *U*_eq_(C). The CF_3_ group showed rotational disorder. For atoms F1A, F2A and F3A the site occupancy factor is 0.288 (17) and for F1B, F2B and F3B the site occupancy factor is 0.712 (17). The disorder was refined using the restraint commands DFIX, ISOR and SIMU.

### Crystal data

**Table:**

C_14_H_9_F_4_NO_2_	*F*(000) = 608
*M**_r_* = 299.22	*D*_x_ = 1.515 Mg m^−^^3^
Monoclinic, *P*2_1_/*c*	Mo *K*α radiation, λ = 0.71073 Å
Hall symbol: -P 2ybc	Cell parameters from 8512 reflections
*a* = 14.223 (2) Å	θ = 1.6–28.5°
*b* = 7.0894 (6) Å	µ = 0.14 mm^−^^1^
*c* = 13.2479 (19) Å	*T* = 296 K
β = 100.910 (11)°	Prism., yellow
*V* = 1311.7 (3) Å^3^	0.72 × 0.34 × 0.07 mm
*Z* = 4	

### Data collection

**Table:**

Stoe IPDS-II diffractometer	2581 independent reflections
Radiation source: fine-focus sealed tube	1261 reflections with *I* > 2σ(*I*)
graphite	*R*_int_ = 0.063
Detector resolution: 6.67 pixels mm^-1^	θ_max_ = 26.0°, θ_min_ = 2.9°
ω scans	*h* = −17→17
Absorption correction: integration (*X-RED32*; Stoe & Cie, 2002)	*k* = −8→8
*T*_min_ = 0.929, *T*_max_ = 0.985	*l* = −16→16
8512 measured reflections	

### Refinement

**Table:**

Refinement on *F*^2^	Primary atom site location: structure-invariant direct methods
Least-squares matrix: full	Secondary atom site location: difference Fourier map
*R*[*F*^2^ > 2σ(*F*^2^)] = 0.057	Hydrogen site location: inferred from neighbouring sites
*wR*(*F*^2^) = 0.133	H atoms treated by a mixture of independent and constrained refinement
*S* = 1.00	*w* = 1/[σ^2^(*F*_o_^2^) + (0.0516*P*)^2^] where *P* = (*F*_o_^2^ + 2*F*_c_^2^)/3
2581 reflections	(Δ/σ)_max_ < 0.001
222 parameters	Δρ_max_ = 0.12 e Å^−^^3^
66 restraints	Δρ_min_ = −0.14 e Å^−^^3^

### Special details

**Table:**

Geometry. All esds (except the esd in the dihedral angle between two l.s. planes) are estimated using the full covariance matrix. The cell esds are taken into account individually in the estimation of esds in distances, angles and torsion angles; correlations between esds in cell parameters are only used when they are defined by crystal symmetry. An approximate (isotropic) treatment of cell esds is used for estimating esds involving l.s. planes.
Refinement. Refinement of F^2^ against ALL reflections. The weighted R-factor wR and goodness of fit S are based on F^2^, conventional R-factors R are based on F, with F set to zero for negative F^2^. The threshold expression of F^2^ > 2sigma(F^2^) is used only for calculating R-factors(gt) etc. and is not relevant to the choice of reflections for refinement. R-factors based on F^2^ are statistically about twice as large as those based on F, and R- factors based on ALL data will be even larger.

### Fractional atomic coordinates and isotropic or equivalent isotropic displacement parameters (Å^2^)

**Table:**

	*x*	*y*	*z*	*U*_iso_*/*U*_eq_	Occ. (<1)
C1	0.6817 (2)	0.1145 (4)	0.8494 (2)	0.0532 (7)	
C2	0.7714 (2)	0.1111 (4)	0.8217 (2)	0.0590 (8)	
H2	0.7757	0.1033	0.7526	0.071*	
C3	0.8530 (2)	0.1190 (4)	0.8947 (2)	0.0609 (8)	
C4	0.8492 (2)	0.1262 (5)	0.9975 (2)	0.0713 (9)	
H4	0.9054	0.1308	1.0465	0.086*	
C5	0.7622 (2)	0.1265 (5)	1.0275 (2)	0.0720 (9)	
H5	0.7595	0.1308	1.0971	0.086*	
C6	0.6781 (2)	0.1207 (4)	0.9551 (2)	0.0578 (7)	
C7	0.5955 (2)	0.1111 (4)	0.7713 (2)	0.0564 (7)	
H7	0.6011	0.1079	0.7025	0.068*	
C8	0.42736 (18)	0.1115 (4)	0.7176 (2)	0.0488 (7)	
C9	0.3428 (2)	0.1172 (4)	0.7535 (2)	0.0626 (8)	
H9	0.3441	0.1207	0.8239	0.075*	
C10	0.2558 (2)	0.1176 (4)	0.6859 (3)	0.0734 (9)	
H10	0.1992	0.1217	0.7114	0.088*	
C11	0.2521 (2)	0.1121 (5)	0.5813 (3)	0.0707 (9)	
H11	0.1941	0.1132	0.5351	0.085*	
C12	0.3369 (2)	0.1050 (5)	0.5484 (2)	0.0666 (8)	
C13	0.4241 (2)	0.1054 (4)	0.6120 (2)	0.0647 (8)	
H13	0.4801	0.1017	0.5855	0.078*	
C15	0.9817 (2)	0.2629 (7)	0.8394 (3)	0.0769 (10)	
N1	0.51254 (17)	0.1122 (3)	0.79347 (16)	0.0539 (6)	
O1	0.59429 (17)	0.1222 (4)	0.98783 (16)	0.0776 (7)	
O2	0.94418 (15)	0.1066 (3)	0.86652 (18)	0.0787 (7)	
F1A	0.9358 (12)	0.389 (2)	0.7861 (18)	0.117 (5)	0.288 (17)
F2A	1.0127 (14)	0.332 (3)	0.9365 (12)	0.132 (6)	0.288 (17)
F3A	1.0594 (14)	0.243 (4)	0.7992 (19)	0.129 (8)	0.288 (17)
F1B	0.9331 (4)	0.3107 (14)	0.7446 (5)	0.115 (2)	0.712 (17)
F2B	0.9765 (5)	0.4159 (9)	0.8941 (7)	0.110 (2)	0.712 (17)
F3B	1.0706 (4)	0.2329 (14)	0.8333 (7)	0.0968 (19)	0.712 (17)
F4	0.33425 (15)	0.0950 (4)	0.44555 (14)	0.1095 (8)	
H1	0.546 (3)	0.113 (6)	0.932 (4)	0.144 (18)*	

### Atomic displacement parameters (Å^2^)

**Table:**

	*U*^11^	*U*^22^	*U*^33^	*U*^12^	*U*^13^	*U*^23^
C1	0.0594 (18)	0.0479 (17)	0.0537 (16)	−0.0005 (15)	0.0148 (14)	0.0009 (15)
C2	0.066 (2)	0.061 (2)	0.0517 (16)	0.0087 (16)	0.0163 (15)	−0.0009 (16)
C3	0.0532 (18)	0.0606 (19)	0.070 (2)	0.0119 (16)	0.0140 (15)	0.0046 (17)
C4	0.067 (2)	0.081 (2)	0.062 (2)	0.0048 (18)	0.0026 (16)	0.0132 (19)
C5	0.074 (2)	0.092 (2)	0.0498 (17)	−0.0028 (19)	0.0107 (16)	0.0077 (18)
C6	0.0584 (19)	0.0612 (19)	0.0551 (18)	−0.0008 (16)	0.0139 (14)	0.0080 (16)
C7	0.068 (2)	0.0540 (19)	0.0500 (17)	−0.0041 (16)	0.0180 (15)	−0.0042 (15)
C8	0.0541 (17)	0.0393 (15)	0.0546 (16)	−0.0024 (14)	0.0145 (14)	0.0010 (14)
C9	0.070 (2)	0.061 (2)	0.0605 (18)	−0.0078 (17)	0.0220 (16)	−0.0063 (16)
C10	0.064 (2)	0.074 (2)	0.086 (3)	0.0009 (18)	0.0242 (18)	−0.006 (2)
C11	0.060 (2)	0.064 (2)	0.083 (2)	−0.0019 (17)	0.0011 (17)	0.0037 (18)
C12	0.076 (2)	0.072 (2)	0.0500 (18)	−0.0008 (18)	0.0055 (16)	0.0092 (17)
C13	0.066 (2)	0.079 (2)	0.0520 (17)	0.0000 (17)	0.0191 (15)	0.0068 (17)
C15	0.055 (2)	0.096 (3)	0.082 (3)	0.006 (2)	0.018 (2)	0.001 (3)
N1	0.0594 (15)	0.0536 (15)	0.0496 (13)	−0.0024 (13)	0.0127 (11)	0.0003 (12)
O1	0.0673 (15)	0.116 (2)	0.0532 (13)	−0.0080 (14)	0.0212 (11)	0.0009 (13)
O2	0.0605 (14)	0.0801 (17)	0.0998 (17)	0.0159 (13)	0.0264 (12)	0.0094 (14)
F1A	0.103 (7)	0.096 (7)	0.160 (10)	0.020 (6)	0.044 (8)	0.026 (7)
F2A	0.128 (9)	0.128 (10)	0.141 (8)	−0.039 (7)	0.030 (6)	−0.039 (7)
F3A	0.115 (10)	0.153 (11)	0.136 (12)	0.006 (7)	0.068 (8)	−0.001 (8)
F1B	0.094 (3)	0.164 (5)	0.083 (3)	−0.015 (3)	0.008 (2)	0.044 (3)
F2B	0.113 (4)	0.086 (3)	0.143 (5)	−0.019 (3)	0.053 (3)	−0.034 (3)
F3B	0.048 (2)	0.137 (4)	0.110 (4)	0.008 (2)	0.025 (2)	0.011 (3)
F4	0.1097 (16)	0.162 (2)	0.0533 (11)	−0.0061 (15)	0.0052 (10)	0.0172 (12)

### Geometric parameters (Å, °)

**Table:**

C1—C2	1.393 (4)	C9—C10	1.383 (4)
C1—C6	1.411 (4)	C9—H9	0.9300
C1—C7	1.447 (4)	C10—C11	1.378 (4)
C2—C3	1.365 (4)	C10—H10	0.9300
C2—H2	0.9300	C11—C12	1.358 (4)
C3—C4	1.374 (4)	C11—H11	0.9300
C3—O2	1.418 (3)	C12—F4	1.358 (3)
C4—C5	1.369 (4)	C12—C13	1.361 (4)
C4—H4	0.9300	C13—H13	0.9300
C5—C6	1.385 (4)	C15—F1A	1.246 (12)
C5—H5	0.9300	C15—F3B	1.299 (6)
C6—O1	1.343 (3)	C15—O2	1.310 (4)
C7—N1	1.268 (3)	C15—F2B	1.315 (5)
C7—H7	0.9300	C15—F3A	1.321 (13)
C8—C9	1.376 (4)	C15—F1B	1.357 (6)
C8—C13	1.392 (4)	C15—F2A	1.369 (11)
C8—N1	1.420 (3)	O1—H1	0.91 (5)
			
C2—C1—C6	118.0 (3)	C10—C11—H11	121.4
C2—C1—C7	120.4 (3)	C11—C12—F4	117.8 (3)
C6—C1—C7	121.7 (3)	C11—C12—C13	124.2 (3)
C3—C2—C1	120.7 (3)	F4—C12—C13	118.0 (3)
C3—C2—H2	119.6	C12—C13—C8	118.4 (3)
C1—C2—H2	119.6	C12—C13—H13	120.8
C2—C3—C4	121.0 (3)	C8—C13—H13	120.8
C2—C3—O2	120.5 (3)	F1A—C15—F3B	119.6 (9)
C4—C3—O2	118.3 (3)	F1A—C15—O2	124.6 (9)
C5—C4—C3	119.7 (3)	F3B—C15—O2	109.3 (6)
C5—C4—H4	120.1	F1A—C15—F2B	69.0 (9)
C3—C4—H4	120.1	F3B—C15—F2B	109.1 (6)
C4—C5—C6	120.6 (3)	O2—C15—F2B	118.6 (4)
C4—C5—H5	119.7	F1A—C15—F3A	103.6 (14)
C6—C5—H5	119.7	F3B—C15—F3A	20.0 (12)
O1—C6—C5	118.6 (3)	O2—C15—F3A	115.9 (14)
O1—C6—C1	121.5 (3)	F2B—C15—F3A	116.6 (14)
C5—C6—C1	120.0 (3)	F1A—C15—F1B	34.5 (8)
N1—C7—C1	122.3 (3)	F3B—C15—F1B	108.4 (5)
N1—C7—H7	118.9	O2—C15—F1B	107.6 (4)
C1—C7—H7	118.9	F2B—C15—F1B	103.5 (5)
C9—C8—C13	118.9 (3)	F3A—C15—F1B	88.4 (11)
C9—C8—N1	116.1 (3)	F1A—C15—F2A	108.5 (10)
C13—C8—N1	125.0 (2)	F3B—C15—F2A	88.4 (8)
C8—C9—C10	120.7 (3)	O2—C15—F2A	96.8 (9)
C8—C9—H9	119.7	F2B—C15—F2A	39.6 (9)
C10—C9—H9	119.7	F3A—C15—F2A	105.7 (12)
C11—C10—C9	120.7 (3)	F1B—C15—F2A	143.0 (10)
C11—C10—H10	119.7	C7—N1—C8	122.9 (2)
C9—C10—H10	119.7	C6—O1—H1	109 (3)
C12—C11—C10	117.2 (3)	C15—O2—C3	117.4 (3)
C12—C11—H11	121.4		
			
C6—C1—C2—C3	−1.8 (4)	C9—C10—C11—C12	0.4 (5)
C7—C1—C2—C3	178.4 (3)	C10—C11—C12—F4	178.5 (3)
C1—C2—C3—C4	1.5 (5)	C10—C11—C12—C13	−0.9 (5)
C1—C2—C3—O2	177.0 (3)	C11—C12—C13—C8	0.7 (5)
C2—C3—C4—C5	−0.4 (5)	F4—C12—C13—C8	−178.6 (3)
O2—C3—C4—C5	−176.0 (3)	C9—C8—C13—C12	−0.1 (5)
C3—C4—C5—C6	−0.3 (5)	N1—C8—C13—C12	179.8 (3)
C4—C5—C6—O1	−179.7 (3)	C1—C7—N1—C8	179.1 (2)
C4—C5—C6—C1	−0.1 (5)	C9—C8—N1—C7	−178.6 (3)
C2—C1—C6—O1	−179.3 (3)	C13—C8—N1—C7	1.4 (4)
C7—C1—C6—O1	0.5 (5)	F1A—C15—O2—C3	−39.7 (14)
C2—C1—C6—C5	1.1 (4)	F3B—C15—O2—C3	169.1 (5)
C7—C1—C6—C5	−179.1 (3)	F2B—C15—O2—C3	43.4 (7)
C2—C1—C7—N1	179.3 (3)	F3A—C15—O2—C3	−170.4 (13)
C6—C1—C7—N1	−0.5 (4)	F1B—C15—O2—C3	−73.4 (6)
C13—C8—C9—C10	−0.3 (5)	F2A—C15—O2—C3	78.4 (11)
N1—C8—C9—C10	179.8 (3)	C2—C3—O2—C15	85.7 (4)
C8—C9—C10—C11	0.1 (5)	C4—C3—O2—C15	−98.6 (4)

### Hydrogen-bond geometry (Å, °)

**Table:**

*D*—H···*A*	*D*—H	H···*A*	*D*···*A*	*D*—H···*A*
O1—H1···N1	0.91 (5)	1.81 (5)	2.618 (3)	146 (4)

## References

[bb1] Calligaris, M., Nardin, G. & Randaccio, L. (1972). *Coord. Chem. Rev.* **7**, 385–403.

[bb2] Cohen, M. D., Schmidt, G. M. J. & Flavian, S. (1964). *J. Chem. Soc.* pp. 2041–2051.

[bb3] Farrugia, L. J. (1997). *J. Appl. Cryst.* **30**, 565.

[bb4] Farrugia, L. J. (1999). *J. Appl. Cryst.* **32**, 837–838.

[bb5] Hadjoudis, E., Vitterakis, M., Moustakali, I. & Mavridis, I. (1987). *Tetrahedron*, **43**, 1345–1360.

[bb6] Şahin, O., Albayrak, C., Odabaşoğlu, M. & Büyükgüngör, O. (2005). *Acta Cryst.* E**61**, o2859–o2861. 10.1107/S0108270105005500 15805639

[bb7] Sheldrick, G. M. (2008). *Acta Cryst.* A**64**, 112–122. 10.1107/S0108767307043930 18156677

[bb8] Stoe & Cie (2002). *X-AREA* and *X-RED32* Stoe & Cie, Darmstadt, Germany.

